# When half is more than the whole: Wheat domestication syndrome reconsidered

**DOI:** 10.1111/eva.13472

**Published:** 2022-09-20

**Authors:** Zvi Peleg, Shahal Abbo, Avi Gopher

**Affiliations:** ^1^ Robert H. Smith Institute of Plant Sciences and Genetics in Agriculture The Hebrew University of Jerusalem Rehovot Israel; ^2^ Sonia and Marco Nadler Institute of Archaeology Tel‐Aviv University Ramat Aviv Israel

**Keywords:** brittle rachis spike, domestication syndrome, seed dispersal, shattering vs. nonshattering spike, wheat domestication

## Abstract

Two opposing models currently dominate Near Eastern plant domestication research. The *core area‐one event* model depicts a knowledge‐based, conscious, geographically centered, rapid single‐event domestication, while the *protracted‐autonomous* model emphasizes a noncentered, millennia‐long process based on unconscious dynamics. The latter model relies, in part, on quantitative depictions of diachronic changes (in archaeological remains) in proportions of spikelet shattering to nonshattering, towards full dominance of the nonshattering (domesticated) phenotypes in cultivated cereal populations. Recent wild wheat genome assembly suggests that shattering and nonshattering spikelets may originate from the same (individual) genotype. Therefore, their proportions among archaeobotanical assemblages cannot reliably describe the presumed protracted‐selection dynamics underlying wheat domestication. This calls for a reappraisal of the “domestication syndrome” concept associated with cereal domestication.

## 1 RECENT EVIDENCE UPSETS THE CURRENT CEREAL DOMESTICATION MODEL

**FIGURE 1:**
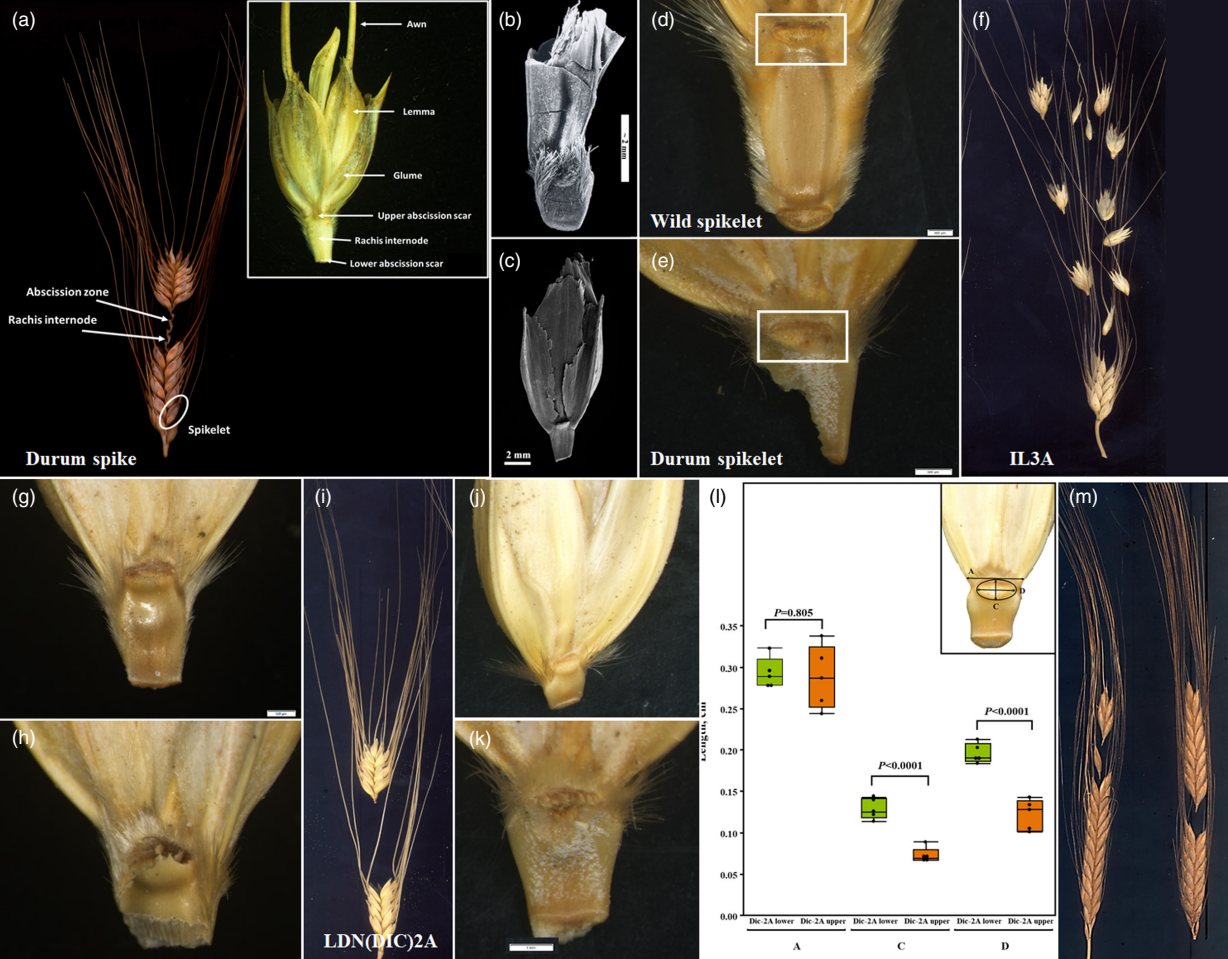
Spike brittleness in wheat. (a) Terminology of the wheat spike organs, depicting the rachis segments and spikelets and a single spikelet in ventral view. Archaeobotanical samples of (b) wild spikelet from the Ohalo II (dated 23,000 years ago) and (c) domesticated spikelet from the A'rugot cave (dated to the second century AD). (d) Wild emmer wheat (*Triticum turgidum* ssp. *dicoccoides*) spikelet with smooth wild abscission scar, and (e) durum wheat (*T. turgidum* ssp. *durum*) spikelet with a jagged break. (f) The phenotype of introgression line (IL)‐3A with intermediate brittle rachis and an abscission scar (g), an upper (smooth scar similar to wild wheat), and (h) bottom (rough edges torn from the nonshattering rachis similar to domesticated durum wheat). (i) The phenotype of wild emmer chromosome substitution line LDN(DIC)2A with an intermediate brittle rachis and an abscission scar of (j) an upper and (k) bottom parts of the spike. (l) Measures of the A (maximal width of the spikelet base, above the scar), D (scar width), and C (scar length) (based on Snir & Weiss, 2014). *p*‐Values represent differences between upper and lower spikelets, *t*‐test (*n* = 6). (m) A representative photo of mature spikes of domesticated emmer (*T. turgidum* ssp. *dicoccum*) cultivars, with quasi‐brittle rachises.

The use of a quantitative approach when describing plant domestication in the Levant (i.e., quantify the archaeobotanical remains and statistically analyse the numbers and frequencies) has long been considered relevant to cereals since, as opposed to legumes (and flax; *Linum usitatissimum*), in the archaeobotanical remains of cereals, it is possible to determine whether a particular cereal is wild (shattering spikes) or domesticated (nonshattering spikes) (Tanno & Willcox, 2006). Biologically, the primary reasoning was that domesticated spikes (e.g., spikelets of nonbrittle/nonshattering cereals) are ill‐adapted to natural settings, whereas under cultivation this very phenotype is of prime value for the farmer (e.g., Harlan et al., 1973). During the last 70 years, domestication research on the Near Eastern cereals wheat (*Triticum* sp.) and barley (*Hordeum spontaneum*) followed the assumption that the abscission scar of wild (i.e., shattering) types of cereals can be distinguished from the threshing marks of domesticated (i.e., nonshattering) cereals among the archaeobotanical remains retrieved from archaeological sites. The rule of thumb was that wild types show smooth abscission scars while domesticated ones show rough scars on the spike axis (Figure 1). Beyond the technicalities discussed over the years concerning the identification of these morphological markers (e.g., Riehl et al., 2013; Tanno & Willcox, 2012), this procedure became the gold standard for archaeobotanists in the process of identifying domesticated cereals (Figure 1a–e) and see fig. 3D in Riehl et al. (2013), for a Neolithic domesticated emmer type from Chogha Golan, Iran.

Quantitative analyses of available archaeobotanical data were presented in the 2000s (e.g., Fuller, 2007; Purugganan & Fuller, 2011; Tanno & Willcox, 2006). With regard to the proportions of shattering versus nonshattering spikelets, the emerging picture showed variability, but a general trend could be detected over time as the nonshattering types became dominant. Not discussed thoroughly, if at all, were questions of whether and how the archaeobotanical remains that were unearthed in Neolithic occupation sites reflect cultivated fields (Abbo et al., 2021). However, archaeobotanical assemblages were used to show how these general trends were reflected in the quantitative depictions of spikelet remains (Fuller et al., 2014), under the (implicit) assumption that the ratio between shattering and nonshattering remains, as documented in archaeological sites (ancient human settlements), correctly represents the genetic structures of the cereal populations that were supposedly cultivated by the Neolithic communities in the Levant. Moreover, the diachronic rise in the proportions of domestic types versus wild types has recently been used as a basis for subdivisions within the (presumed) long sequence of predomestication cultivation (Box 1; Fuller et al., 2018). We recently addressed these issues and questioned the agronomic, as well as the archaeological logic of the above assumption (Abbo & Gopher, 2020; Abbo et al., 2021).

BOX 1 **Predomestication cultivation**

*Predomestication cultivation* (PDC) denotes wild‐plant cultivation prior to domestication. While attempting to better understand cultivation, Hillman and Davies (1999) coined this term when working with cereal archaeobotanical remains at the site of Tell Abu Hureyra. *Predomestication cultivation* (leading to morphological domestication) is a phase during which the managed plant stocks possess *WT* phenotypes, as recruited from the wild. Hillman and Davies (1999) attempted to provide experimental evidence for PDC. Interestingly, in their study on einkorn wheat, they concluded that its domestication could have been achieved within 20–200 years (Hillman & Davies, 1990a, 1990b, 1992, 1999)—meaning that PDC was viewed as a relatively short phase prior to domestication and domestication itself was rapid. This construct later evolved into a concept implicitly indicating a phase in the millennia‐long human‐plant relationship that led to domestication (see Abbo & Gopher, 2020). Recently, the PDC concept become almost synonymous with a protracted, millennia‐long process of slowly evolving domesticated plants (e.g., Fuller et al., 2014).

One of the important studies underlying the wide use of the term PDC employed the archaeobotany of the late Natufian layer 1 at Tell Abu Hureyra, Syria (e.g., Hillman, 2000; Hillman & Davies, 1990b, 1992, 1999; Hillman et al., 1989). Four aspects were found useful by Hillman (2000) and by Hillman et al. (2001) when they proposed PDC in the Natufian (13,000 calibrated years before present) layer 1 of Tell Abu Hureyra; (i) the presence of seeds of the so‐called weeds of cultivation; (ii) the geographic displacement of particular food plants; (iii) shifts in use‐wear patterns on flint sickle blades; and (iv) a change in grain size. These aspects remain central to the issue but are not reviewed here. Suffice is to say that detailed scrutiny of these aspects raises many difficulties, rendering cultivation, let alone PDC difficult to support (Abbo & Gopher, 2020; Gopher et al., 2021).

In light of the recently deciphered wild emmer wheat [*Triticum turgidum* ssp*. dicoccoides* (Körn.) Thell.] genome (Avni et al., 2017) and its possible implications *vis‐à‐v*is the selection of cereal (wheat) phenotypes for domestication, we herein address the fundamental set of ideas, practices, and assumptions concerning Near Eastern cereals domestication. We question the validity both of identifying nonshattering versus shattering types as the pre‐eminent “Domestication Syndrome” trait, and of using their respective proportions in the archaeobotanical remains of cereals to promote a protracted‐domestication model and as a reflector of a long predomestication‐cultivation phase (see Box 1).

## 2 GENETIC CONSIDERATIONS PERTAINING TO FREQUENCY OF SPIKE SHATTERING IN WHEAT

### 2.1 Genetic control of the spike brittleness trait

Wild emmer wheat, the direct progenitor of domesticated wheat, is characterized, like other members of the *Triticeae* tribe, by a brittle inflorescence along its rachis that shatters spontaneously into dispersal units (spikelets) upon maturity. The arrow‐like shape of the spikelets facilitates their penetration through surface litter into the soil, providing appropriate conditions for future germination while at the same time minimizing seed predation by ants and rodents. These adaptive features confer evolutionary significance on the brittle rachis (Br) trait. The results of genetic and cytogenetic analyses show that the Br character in wheat is dominant (Love & Craig, 1919), polygenic, and controlled by several loci on homoeologous groups 2, 3, and 4 (Avni et al., 2017; Dvorak et al., 2006; Nalam et al., 2006; Nave et al., 2021; Peleg et al., 2011; Peng et al., 2003; Watanabe, 2005; Watanabe & Ikebata, 2000; Zeng et al., 2020; Zhao et al., 2019).

The reduction in spike disarticulation at maturity of (domesticated) grain crops evolved independently, often by the same gene(s) (and/or even the same mutation), and reflects a convergent morphological adaptation to artificial meticulous human selection (reviewed by Dong & Wang, 2015; Maity et al., 2021; Olsen & Wendel, 2013; Sakuma et al., 2011). In barley, a loss‐of‐function mutation in one of the two complementary dominant genes, *Brittle rachis 1* (*Btr1*) and *Btr2* on the short arm of chromosome 3H (Komatsuda & Mano, 2002) resulted in nonshattering spikes (Pourkheirandish et al., 2015). Recently, Avni et al. (2017) and Pourkheirandish et al. (2018) showed that loss‐of‐function mutations in the *Brittle rachis 1* gene (*Btr1*)‐*A* and *TtBtr1‐B* in the A and B subgenomes, respectively, result in nonshattering spikes in all domesticated wheat (einkorn, domesticated emmer, durum, and bread wheat). Notably, the nonbrittle rachis phenotype of domesticated einkorn (*T. monococcum*) is a consequence of another nonsynonymous mutation in the *TmBtr1‐A* gene (Pourkheirandish et al., 2018). Zeng et al. (2020) showed that all wild *Triticeae* species exhibiting disarticulation above the rachis nodes carry a copy of the *Btr1* gene.

Using wild emmer wheat (acc. Zavitan) near‐isogenic lines, Avni et al. (2017) demonstrated that only one functional allele (*Btr‐A* or *Btr‐B* on chromosomes 3A and 3B, respectively), on a domesticated durum wheat background (*cv*. Svevo), results in an intermediate phenotype (brittle upper part of the spike and nonbrittle lower spike section) (see fig. 3 in Avni et al., 2017; Figure 1f–h). However, the mutation type is different between chromosomes 3A and 3B, with a 2‐bp deletion on *Btr‐A* leading to a premature termination codon and a 4‐kb insertion (539 bases from the start codon), resulting in a longer C‐terminus protein sequence in *Btr‐B*. Moreover, while a mutation in *Btr2* causes a non‐Br spike in barley, in wheat no variation was observed in this locus (Avni et al., 2017; Pourkheirandish et al., 2015).

The polygenic nature of spike rachis brittleness corresponds with additional genomic regions affecting this trait found in barley (i.e., chromosomes 5H and 7H; Komatsuda et al., 2004), einkorn (i.e., chromosomes 4A and 7A; Pourkheirandish et al., 2018), and wheat [i.e., chromosomes 2A (Peleg et al., 2011) and 4A (Dvorak et al., 2006)], as well as possible interaction with other genes such as the free‐threshing gene *Q* (a member of the AP2 class transcription factors; Simons et al., 2006), may suggest that a partly brittle phenotype may occur via other pathways and not necessarily by factors carried on chromosome group 3. Likewise, various crosses in barley using different combinations of *Br* alleles resulted in a range of rachis brittleness (Fernández‐Calleja et al., 2020). Notably, while the spike of domesticated emmer [*T. turgidum* ssp. *dicoccum* (Schrank) Thell.] is less brittle than that of wild emmer, upon maturation of the former the slightest mechanical stress causes its disarticulation. Moreover, certain traditional emmer cultivars still have quasi‐brittle rachises [e.g., from Ethiopia (Belay & Furuta, 2001), Russia and Italy (Watanabe, 2005)] (Figure 1m).

### 2.2 Effects of mutation in Btr loci on fitness and visibility of their respective phenotypes

**FIGURE 2:**
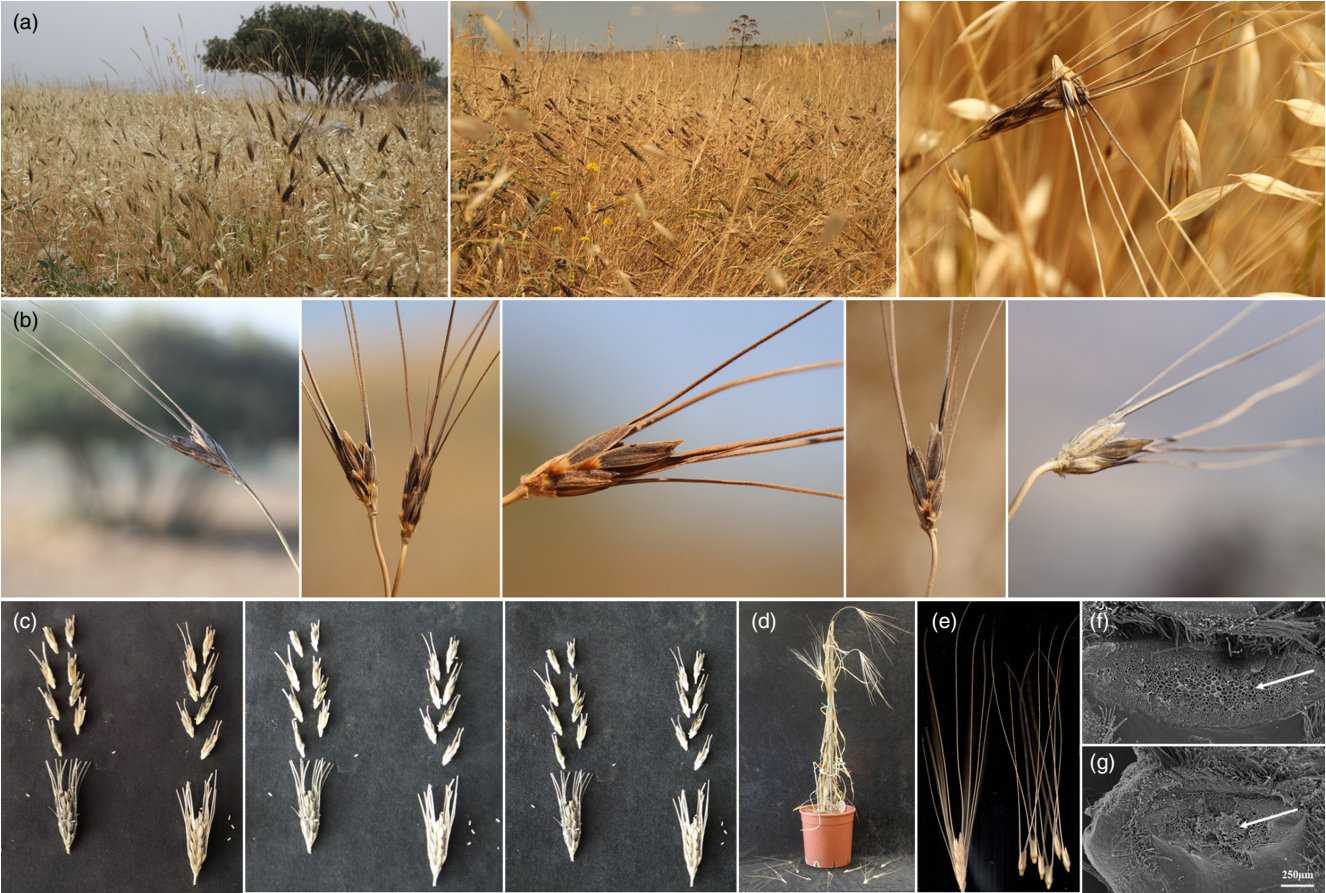
Spike‐shattering patterns in wild emmer. (a) Wild emmer wheat (*Triticum turgidum* ssp. *dicoccoides*) plants in their natural habitats in Israel, with mature disarticulating spikes. (b) Examples of wild emmer spikes after shattering, with 2–5 remaining spikelets (photos taken more than 2 months after full maturity). (c) Examples of half‐spike brittleness wild emmer spikes of plants grown under controlled conditions (collected in the Arbel nature reserve and the Mt. Gilboa habitats, Northern Israel), and (d) mature near‐isogenic line (NIL) of wild emmer wheat accession Zavitan with introgressed domesticated alleles of *Btr‐A* (chromosome 3A), and (e) spikes with a half‐brittle phenotype. SEM image of an abscission scar of an (f) upper and (g) lower spikelets. The upper image (f) confirming that the NILs have smooth scars similar to those of wild emmer Zavitan (i.e., BR phenotype) and lower image (g) confirming that the NILs have rough edges scars similar to those of durum wheat. White arrow points to the smooth or rough edges scar associated with the shattering versus nonshattering phenotype.

In natural ecosystems, wild‐plant fitness is strongly influenced by propagule dispersal, which determines the locations where seeds, and subsequently seedlings, will be established. Theoretically, therefore, plants will enjoy higher fitness if a higher proportion of seeds is dispersed into sites where offspring are predicted to have a high probability of survival relative to random sites. Kamm (1974) reported an encounter of a single plant with a nonbrittle rachis within a wild emmer wheat population on Mount Gilboa in Israel. Over the past 20 years we have repeatedly surveyed wild emmer stands in Israel in an attempt to identify such individuals but to no avail. Indeed, owing to its low fitness, the chances of such a phenotype surviving in nature over several generations is rather low. It was suggested that such nonbrittle mutations are likely to be eliminated quickly upon formation, especially under conditions of heavy grazing (Kamm, 1974). However, the finding that a single mutation in one of the two orthologous *Btr1* genes confers brittleness of only half the spike calls for a reappraisal of the classical conventions of wheat evolution (Abbo et al., 2021; Hillman & Davies, 1990a). During some of our field surveys, we indeed observed (and sampled seeds of) populations having a phenotype of partial shattering spikes (Figure 2a,b; Video S1), which may suggest that hunter‐gatherers were able to visually recognize and appreciate the potential of such a “half‐spike” trait. Notably, growing these accessions in controlled conditions showed a similar phenotype of half‐spike brittleness (Figure 2c). Moreover, we have developed two introgression lines of wild emmer wheat accession Zavitan, with the domesticated allele of *Btr‐A* or *Btr‐B* (chromosomes 3A and 3B, respectively) that present similar half‐spike brittleness phenotype (Figure 2d,e). Scanning electron microscopy (SEM) confirmed smooth abscission scars typical of wild emmer spikelets in the scar tissues of spikelets from the upper rachises (Figure 2f), whereas the lower nonbrittle spikelets had rough abscission scars similar to those of domesticated wheat (Figure 2g).

A stable half‐spike brittleness, mediated by a mutation in only one of the abovementioned Br genes, may extend the survival (and, to some extent, facilitate the propagation) of such naturally occurring mutants in native populations. Longer‐term survival of half‐brittle genotypes in natural stands may have increased their visibility to humans who were surveying wild populations in search for their first seed stocks, compared with the expected very low visibility of the extremely rare, fully nonbrittle type in the wild. Likewise, Brandolini and Heun (2019) reported that truly wild einkorns from the Karacadağ region (Turkey) contain patches of naturally emerging mutants with semi‐brittle rachis. Taking into consideration the present patchy nature of wild emmer distribution across the Near East, as well as its semi‐isolation, its small size (relative to pre‐agrarian times), and the frequency rate of new mutations in eukaryotes (~1 × 10^−8^/base pair/generation; Baer et al., 2007), the chances of observing fully nonbrittle rachis spikes (plants with two mutations in both *Btr‐A* and *Btr‐B* over a single human generation) are low. In general, deleterious variants occur at higher frequencies than those conferring beneficial effects (Felsenstein, 1974). Moreover, in natural populations, we expect to observe fewer deleterious mutations (such as nonbrittle rachis variants) than in anthropogenic populations, where natural selection pressures are relaxed (Agrawal & Whitlock, 2012; Harlan et al., 1973; Moyers et al., 2018). However, if—as mentioned above—a single mutation in one of the *Btr* loci can produce a quasi‐stable (even if small) population, the chances of people having observed such stocks in the field during their lifetime would have been much higher. In other words, it seems reasonable to suggest that such a single mutation (in wild wheat, for example) is not necessarily as deleterious as usually portrayed in the literature (Purugganan & Fuller, 2011) but may rather be considered a more tolerable mutation in nature.

## 3 SIMPLISTIC INTERPRETATIONS OF THE ARCHAEOBOTANICAL RECORD

Considering the possibility that the first stocks of wheat seed corn were sampled from wild populations in which quasi‐shattering individuals (i.e., bearing such a single mutation) were present (at certain frequencies) and that a second mutation occurred in the course of wheat evolution under domestication, this “half‐shattering” phenotype significantly complicates interpretation of archaeobotanical spike remains. This is because the proportion of brittle spikelets (relative to nonbrittle ones) in the archaeological record may have resulted from various human activities (see fig. 12 in Hillman & Davies, 1990b), as well as from intra‐ and interannual climatic fluctuations and seasonal conditions. For example, harvesting early in the season (before shattering begins) will result in a mix of brittle and nonbrittle spikelets, whereas harvesting after shattering, i.e., late in the season (for reasons such as time limitations, or location of the stand(s) relative to occupation sites) may increase the frequency of nonbrittle spike remains. Ecological conditions (e.g., soil type, precipitation, temperature, relative humidity) are likely to affect the dynamics of plant maturation processes, including the rate of shattering from the upper fraction of spikes having a single *Btr* mutation. The Fertile Crescent region is characterized by wide inter‐annual and seasonal fluctuations in soil moisture, thereby influencing the relative fitness of various phenological traits (such as germination, flowering time, and maturation period) (See fig. 5 in Peleg et al., 2008) Under such environmental conditions, harvest operations may shift between years even within the same habitats and are therefore likely to change the proportions of brittle versus nonbrittle spikelets in any given year. An example of such a possible shift in maturity (that may have necessitated changes in past harvest timing) was observed by us in April 2021, when following a few days of a heatwave, extensive stands of “half spikes” were recorded on Mount Gilboa, Israel (Figure 2a,b).

## 4 DISCUSSION

Near Eastern archaeobotanical literature shows that cereals were always central to plant domestication research and that the relatively straightforward procedure of identifying them as wild or domesticated (i.e., shattering or nonshattering) provides valuable quantitative data. Seeds of barley were recorded in the middle Paleolithic (60–50 KYA) Kebara cave (Lev et al., 2005) and cereals (wheat, barley, and oats) in significant numbers appear in the early Epipaleolithic (23 KYA) site of Ohalo II (Snir et al., 2015). Shattering and nonshattering cereals continue to appear in archaeobotanical assemblages throughout the Neolithic period; however, their proportions should not be interpreted simplistically. Changing frequencies reported for wild and domestic archaeoforms were fundamental in formulating certain plant domestication models, and the increasing proportion of nonshattering spike remains is considered evidence of a protracted time frame for domestication (e.g., Douché & Willcox, 2018; Fuller et al., 2012, 2018; Tanno & Willcox, 2006; Weiss et al., 2006; Willcox, 2013), in accordance with the assumptions of the protracted‐autonomous model (tab. 1 in Abbo & Gopher, 2020). The millennia‐long predomestication cultivation construct was aimed at portraying the prolonged process of plant domestication based (among other arguments) on the observed proportions of shattering versus nonshattering in cereal remains (Fuller et al., 2018).

However, Avni et al. (2017) findings call for a reappraisal of the classical wheat domestication dogma, as both the smooth abscission scar and the rough (“domesticated”) phenotype may appear on the very same spikes (at upper and lower spike internodes, respectively), suggesting that this trait cannot reliably distinguish between a wild emmer population and domesticated wheat. Attempts were made to differentiate between domesticated and wild forms of cereals in terms of spikelet morphometric characteristics (Snir & Weiss, 2014), but even this approach cannot safely define the distinction (Figure 1i–l). In other words, neither the archaeobotanical proportions of brittle versus nonbrittle (nonshattering) spikelets nor their disarticulation scar dimensions can be simply used to reconstruct ancient selection processes (Purugganan & Fuller, 2011) under the assumptions of the protracted‐domestication model (Fuller, 2007; Fuller et al., 2014). Likewise, presenting the frequency of shattering relative to nonshattering remains along diachronic axes cannot safely delimit the time frame attributed to Near Eastern domestication (*à la* Tanno & Willcox, 2006), and therefore cannot possibly support a protracted‐domestication model.

Admittedly, a nonshattering spike is a powerful diagnostic trait for distinguishing wild from domestic cereals. Here, we take issue only with the use of the shattering/nonshattering proportion (over time) as a descriptor of domestication and for delineating its duration (e.g., Abbo et al., 2021). Spikelet remains survive after charring in archaeological sites; these remains may be analysed. The interpretation of the charred spike (and spikelets) remains may give rise to protracted (e.g., Tanno & Willcox, 2006) or rapid (e.g., Tzarfati et al., 2013) models of domestication. It seems that the seed biology of the Near Eastern grain legumes may provide more reliable clues for the pace of domestication (e.g., Abbo & Gopher, 2017; Abbo et al., 2009) due to their strong wild‐type seed dormancy. Charred legumes seeds from archaeological sites cannot be diagnosed as dormant or free germinating, since this is a physiological rather than a morphological trait. Moreover, experimental data vis‐à‐vis legumes biology has shown that no profitable cultivation is possible unless a free germinating mutant is available (e.g., Abbo et al., 2011; Ladizinsky, 1987, 1989). We contend, much in the spirit of Ladizinsky (1987), that for legumes the case was “domestication before cultivation” rendering reconstructions involving many generations of predomestication cultivation irrelevant (see also Abbo et al., 2011). Thus, legume biology and experimental data indicate rapid domestication more reliably than a narrative based on an interpretation of cereals spike remains (and their proportions) from archaeological sites. Given rapid grain legume domestication, we see no reason to assume a protracted process for cereals as part of the Near Eastern founder crops.

## 5 CONCLUDING REMARKS

The importance of our suggestion to re‐consider the conventions of shattering versus nonshattering archaeobotanical remains becomes evident while recalling two recent papers that describe plant domestication in terms of process philosophy (Bogaard et al., 2021) or a “landscape‐level process” (Allaby et al., 2021, 2022) in which plants have adapted themselves to human environmental manipulations over evolutionary time scales starting as early as 25–20 KYA, including an element of unconsciousness (on behalf of the human agent). The data, in support of this new reconstruction, are the proportions of shattering versus nonshattering cereals in archaeobotanical remains (discussed above) and/or diachronic changes in seed sizes that are better viewed as a (post domestication) crop evolution (improvement) trait rather than as a reliable domestication descriptor (sensu Abbo et al., 2014).

## CONFLICT OF INTEREST

The authors declare no conflict of interest.

## Supporting information


Video S1

## Data Availability

There is no data associate with the prespective article.
